# Mixed-reproductive strategies, competitive mating-type distribution and life cycle of fourteen black morel species

**DOI:** 10.1038/s41598-017-01682-8

**Published:** 2017-05-04

**Authors:** Xi-Hui Du, Qi Zhao, En-Hua Xia, Li-Zhi Gao, Franck Richard, Zhu L. Yang

**Affiliations:** 1 Key Laboratory for Plant Diversity and Biogeography of East Asia, Kunming Institute of Botany, Chinese Academy of Sciences, Kunming, China; 2 Plant Germplasm and Genomics Center, Germplasm Bank of Wild Species, Kunming Institute of Botany, Chinese Academy of Sciences, Kunming, Yunnan 650201 China; 3 UMR 5175 CEFE, INSERM, Campus CNRS, F-34293 Montpellier, France

## Abstract

*Morchella* species are well known world-round as popular and prized edible fungi due to their unique culinary flavor. Recently, several species have been successfully cultivated in China. However, their reproductive modes are still unknown, and their basic biology needs to be elucidated. Here, we use the morel genome information to investigate mating systems and life cycles of fourteen black morel species. Mating type-specific primers were developed to screen and genotype ascospores, hymenia and stipes from 223 ascocarps of the 14 species from Asia and Europe. Our data indicated that they are all heterothallic and their life cycles are predominantly haploid, but sterile haploid fruiting also exists. Ascospores in all species are mostly haploid, homokaryotic, and multinuclear, whereas aborted ascospores without any nuclei were also detected. Interestingly, we monitored divergent spatial distribution of both mating types in natural morel populations and cultivated sites, where the fertile tissue of fruiting bodies usually harbored both mating types, whereas sterile tissue of wild morels constantly had one MAT allele, while the sterile tissue of cultivated strains always exhibited both MAT alleles. Furthermore, *MAT1-1-1* was detected significantly more commonly than *MAT1-2-1* in natural populations, which strongly suggested a competitive advantage for *MAT1-1* strains.

## Introduction

As iconic famous edible fruiting bodies, true morels, belonging to the genus *Morchella*, have been highly appreciated, prized and marketed worldwide since antiquity due to their texture and unique scent^1^. Dramatically increased consumption and the rising market led to over-harvest of wild morel resource over-harvest^2^. Recently, except *M. rufobrunnea* ^3^, several other morel species, such as *M. sextelata* (unpublished)*, M. eximia* (unpublished) *and M. importuna* ^4^ can now be successfully cultivated in China, which greatly alleviates the market pressure. However, artificial cultivation, despite under optimized conditions, faces some big problems, such as unstable production, which has brought serious economic loss to the growers in China and urgently needs to be addressed. Fruiting is a crucial step in the fungal life cycle and occurs by either outcrossing or selfing^5^. Therefore, determining the reproductive strategies and mating systems of morels has high economical relevance for cultivation and harvesting. However, shedding light on the mechanisms involved in the reproduction of *Morchella* spp. is a challenging task. Thus far, among morel studies, most of which focused on fungal species richness and distribution of morels, only several were slightly related with the reproductive modes of *Morchella* species^2, 6–8^. The weak research progress in this aspect was attributed, on one hand, to the previous confused taxonomy, on the other hand, to the absence of molecular information on MAT genes of morels, because of the low level of conservation among mating type (MAT) genes in ascomycetes and the absence of genomic sequence.

MAT genes are master regulatory loci controlling sexual reproduction and development in fungi^5^. Heterothallic fungi, namely obligately outcrossing fungi, are self-sterile and usually require the participation of the opposite mating type partner to reproduce. The genes of opposite mating types are located on the same chromosomal locus but are highly divergent and nonhomologous, respectively encoding the alpha-box domain protein (*MAT1-1-1*) and the high mobility group (HMG) protein (*MAT1-2-1*)^9^. Conversely, homothallic fungi are self-fertile and can complete the sexual cycle without a mating partner. Typically a single homothallic strain harbors both MAT genes (linked or unlinked) in the same haploid nucleus^9, 10^. Over the past several years, the advent of molecular techniques, especially genome sequencing, now paves the way for making a significant leap forward in comprehending the life cycles and reproductive modes of *Morchella* species.

In this study, we used the genome sequences of morels to reveal the reproductive modes of black morel species. Our objectives were: (1) to infer their phylogeny and genetic diversity of the *MAT1-1-1* and *MAT1-2-1* genes, (2) to illuminate the reproductive modes of fourteen black morels species and (3) to evaluate both mating types distribution segregation within populations and in ascomatas, and their biparental roles partition. In order to answer these questions, 223 samples belonging to fourteen black morel species were analyzed here, covering wild and cultivated samples, which were assessed by analyzing the presence of both mating types: *MAT1-1-1* and *MAT1-2-1*. All of these results will help to elucidate morel biology and are also of considerable practical impact for optimizing cultivation techniques and increasing production in morels fields.

## Results

### Searching for mating type genes in the Morel genome

The *M. eximia* genome sequence has been conducted in Kunming Institute of Botany, CAS recently. Its database was investigated for the presence of orthologs of ascomycetes MAT genes using BLASTN and TBLASTX similarity searches. First, we respectively chose *Tuber melanosporum* (ADU56595) and *T. borchii* (AIU38078) to blast Alpha-box and HMG-containing sequences in *Morchella* genome. Then we revealed one *MAT1-1-1* and one *MAT1-2-1* which we confirmed by blast to Genbank, resepctively best matching with *T. indicum* (AHE80940, AHE80941), *Stagonosporopsis chrysanthemi* (AHY81336) and *Penicillium kewense* (CBY44653).

### Genetic diversity of MAT idiomorphs and phylogenetic analysis

Both *MAT1-1-1* and *MAT 1-2-1* were successfully amplified and sequenced from the fourteen species. The length of *MAT1-1-1* and *MAT1-2-1* genes respectively varied from 729 to 736 bp and from 398 to 408 bp among these fourteen species, and respectively contained two exons and one intron, and three exons and two introns. Though the length of both genes among these species varied, the protein length they translated was respectively conserved at 189 amino acids and 89 amino acids.

The nucleotide sequences alignment of *MAT1-1-1* included a total of 744 sites after trimming and contained 191 parsimony-informative sites and three singleton variable sites. Its estimated nucleotide diversity (p) was 0.0488 with 191/744 (25.67%) variable nucleotide sites. The *MAT1-2-1* alignment consisted of a total of 411 sites after trimming and included 121 parsimony-informative sites, no singleton variable sites. It had an estimated nucleotide diversity (p) of 0.0574 with 121/411 (29.44%) variable nucleotide sites.

**Figure 1:**
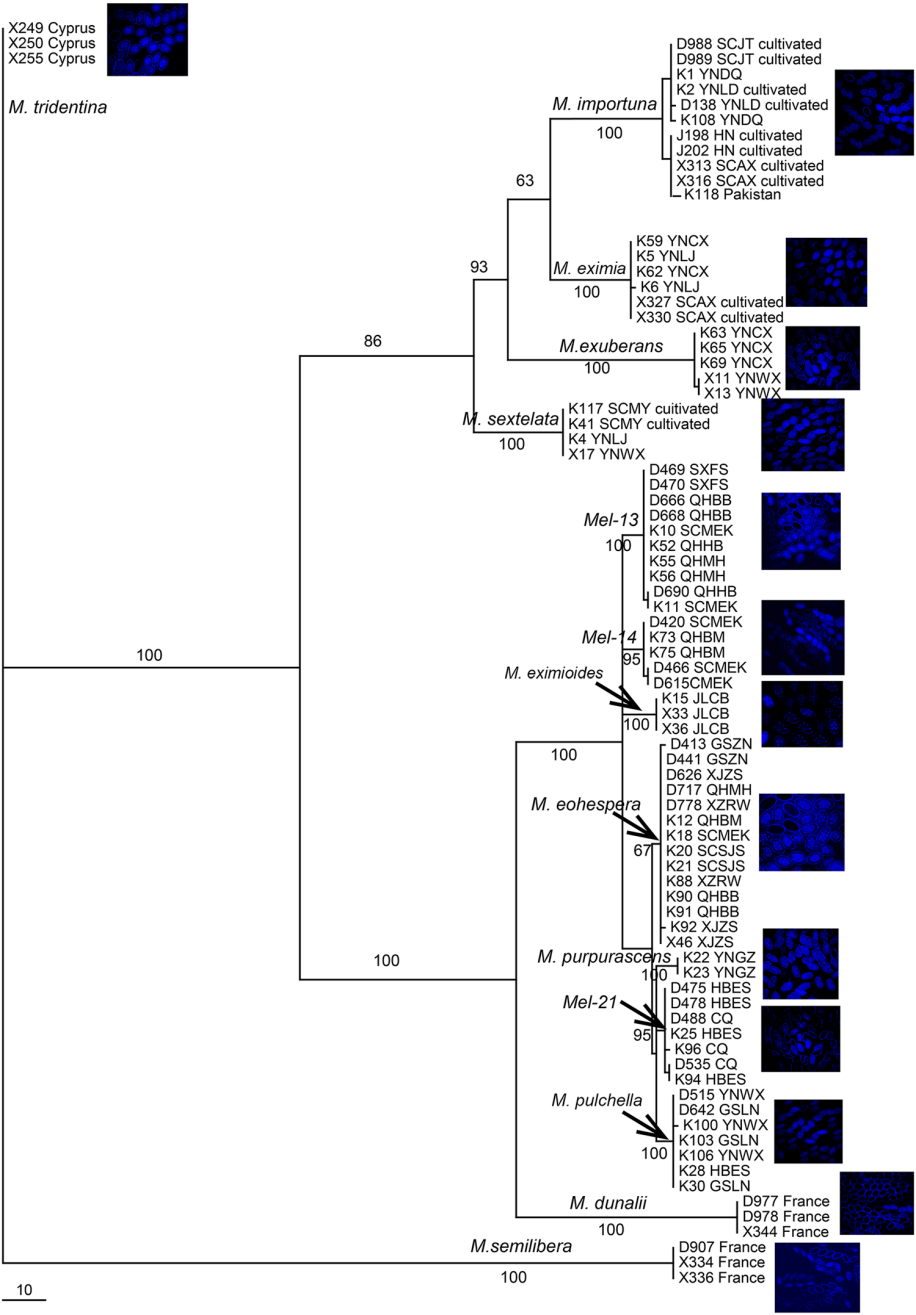
Phylogenetic analyses of 83 collections of fourteen black morel species based on the combined dataset totaling 1155 bp of *MAT1-1-1* and *MAT1-2-1*. Bootstrap values lower than 50% are not shown. The pictures shown beside each species branch are DAPI-staining of ascospores nuclei.

Maximum parsimony phylogenetic analyses were performed with three matrices, viz., *MAT1-1-1* sequences, *MAT1-2-1* sequences, and the combined two-gene sequences. The phylogenetic trees constructed with each of the three matrices had similar topological structures. Herein, only the phylogenetic tree of their combined matrix is shown (Fig. 1). In this phylogenetic tree, each species could be distinguished and grouped together with high bootstrap support respectively as a monophyletic clade (Fig. 1).

### Even mating types ratio of single spore strains in fourteen species

Single ascospores were isolated from the fourteen species, and cultivated as reported in the material and methods section. DNA was successfully extracted from cultures of the single spores, of which the presence and distribution of mating type genes of single spores in each ascomata were assessed using a PCR approach. Each single spore always showed only a single mating type, either the *MAT1-1-1* or the *MAT1-2-1* gene. No spores harboring both mating types were found. The ratio of *MAT1-1-1*:*MAT1-2-1* of single ascopsores in each species was around 1:1 and consisted with the null hypothesis (no deviation from a theoretical segregation ratio of 1:1, *MAT1-1-1*:*MAT1-2-1*) after Fisher’s exact test (Table 1). Therefore, the two mating types occurred equally among ascospores of a single ascomata, and the reproductive modes of these fourteen species were assumed to be heterothallic.

**Table 1:** Spatial segregation of mating types in ascomatas, MAT ratios of single spores and nucleotide diversity of *MAT1-1-1* and *MAT1-2-1* in each species.

Species/N	Site	N	Wild/Cultivated	Year	Spatial segregation types^##^ of MAT/N	*MAT1-1-1*:*MAT1-2-1*in maternal tissue* (*P* value**)	*MAT1-1-1*:*MAT1-2-1*of single spores (*P* value**)	^&^Pi of *MAT1-1-1*/Pi of *MAT1-2-1*
*M. tridentina*/5	Cyprus	5	W	2015	II/5	1.5:1(*P*:1)	24:26 (*P*:1)	0/0
*M. semilibera*/5	France	5	W	2015	II/5	1.5:1 (*P*:1)	25:25 (*P*:1)	0/0
*M. sextelata*/4	Lijiang^#^	1	W	2007	II/1	4:1 (*P*:1)	25:25 (*P*:1)	0/0
	Weixi^#^	1	W	2015	II/1			
	Mianyang^#^	2	C	2015	I/1, II/1			
*M. eximia*/25	Lijiang^#^	3	W	2007	II/3	2.3:1 (*P*:1)	26:24 (*P*:1)	0.00046/0
	Chuxiong^#^	6	W	2014	I/1, II/5			
	Weixi^#^	11	W	2015	II/10, III/1			
	Mianyang^#^	5	C	2016	I/3, II/2			
*M. exuberans*/20	Chuxiong^#^	18	W	2014	I/3, II/14	1.6:1(*P*:1)	25:25 (*P*:1)	0/0
	Weixi^#^	2	W	2015	II/2			
*M. importuna*/39	Deqin^#^	5	W	2007	II/2, III/3	1.2:1 (*P*:1)	25:25 (*P*:1)	0.00189/0.00045
	Mianyang^#^	1	W	2015	I/1			
	Pakistan	1	W	2015	II/1			
	Chengdu^#^	16	C	2014	I/12, II/3, III/1			
	Nanyang^#^	8	C	2016	I/6, II/2			
	Mianyang^#^	8	C	2016	I/5, II/3			
*Mel*-13/20	Haibei^#^	6	W	2012	II/5, III/1	1.8:1 (*P*:1)	27:23 (*P*: 0.841)	0/0.00087
	Aba^#^	2	W	2009	II/2			
	Haidong^#^	2	W	2012	I/2			
	Lvliang^#^	5	W	2009	II/5			
	Haibei^#^	5	W	2012	II/5			
*Mel*-14/18	Aba^#^	6	W	2009	II/6	1.8:1 (*P*:1)	25:25 (*P*:1)	0.00082/0
	Guoluo^#^	12	W	2012	I/4, II/8			
*M. eximioides*/20	Changbai^#^	20	W	2010	II/18, III/2	5.7:1 (*P*:0.471)	24:26 (*P*:1)	0/0
*M. eohespera*/20	Aba^#^	4	W	2009	II/3, III/1	2.3:1 (*P*:1)	26:24 (*P*:1)	0.00039/0
	Enshi^#^	1	W	2010	III/1			
	Haibei^#^	2	W	2012	II/2			
	Changdu^#^	6	W	2012	II/6			
	Zhaosu^#^	3	W	2009	II/3			
	Gannan^#^	4	W	2009	II/4			
*M. purpurascens*/2	Deqin^#^	2	W	2010	II/2	2:0 (*P*:1)	24:26 (*P*:1)	
*Mel*-21/19	Enshi^#^	9	W	2010	I/2, II/7	1.3:1 (*P*:1)	26:24 (*P*:1)	0.00104/0
	Chongqing^#^	10	W	2010	II/10			
*M. dunalii*/5	France	5	W	2015	—^&&^	—^&&^	25:25 (*P*:1)	0/0
*M. pulchella*/21	Enshi^#^	1	W	2010	II/1	1.4:1 (*P*:1)	25:25 (*P*:1)	0/0.00070
	Longnan^#^	12	W	2004	II/12			
	Weixi^#^	8	W	2010	I/3, II/5			

^#^These sites were in China.

^##^Hymenia and stipes were respectively represented as fertile and sterile tissue. I: both of MAT were found in both hymenia layers and stipes; II: both MAT were detected in hymenia layers, but only one mating type, either *MAT1-1-1* or *MAT1-2-1*, in stipes; III: only one MAT was found in these ascocarps, without ascospores after microscopic observation.

*The sterile tissue was defined as maternal tissue here, due to its possible duty of raising the whole ascomata.

**P value was generated by Fisher’s exact test using SPSS10.0 software.

^&^Pi: nucleotide diversity analyzed by DnaSP software.

^&&^Authors only got some part from the hymenia of samples, no stipes, so, the MAT distribution in stipes of *M. dunalii* could not be analyzed.

Nuclei analysis of ascospores from these fourteen species were performed based on DAPI-staining methods, which indicated most of ascospores in each species are haploid homokaryotic multinuclear, but some aborted ascospores without nuclei were also found in each species (Fig. 1).

### Uneven spatial segregation of mating types in fertile and sterile tissue of wild ascocarps

**Figure 2:**
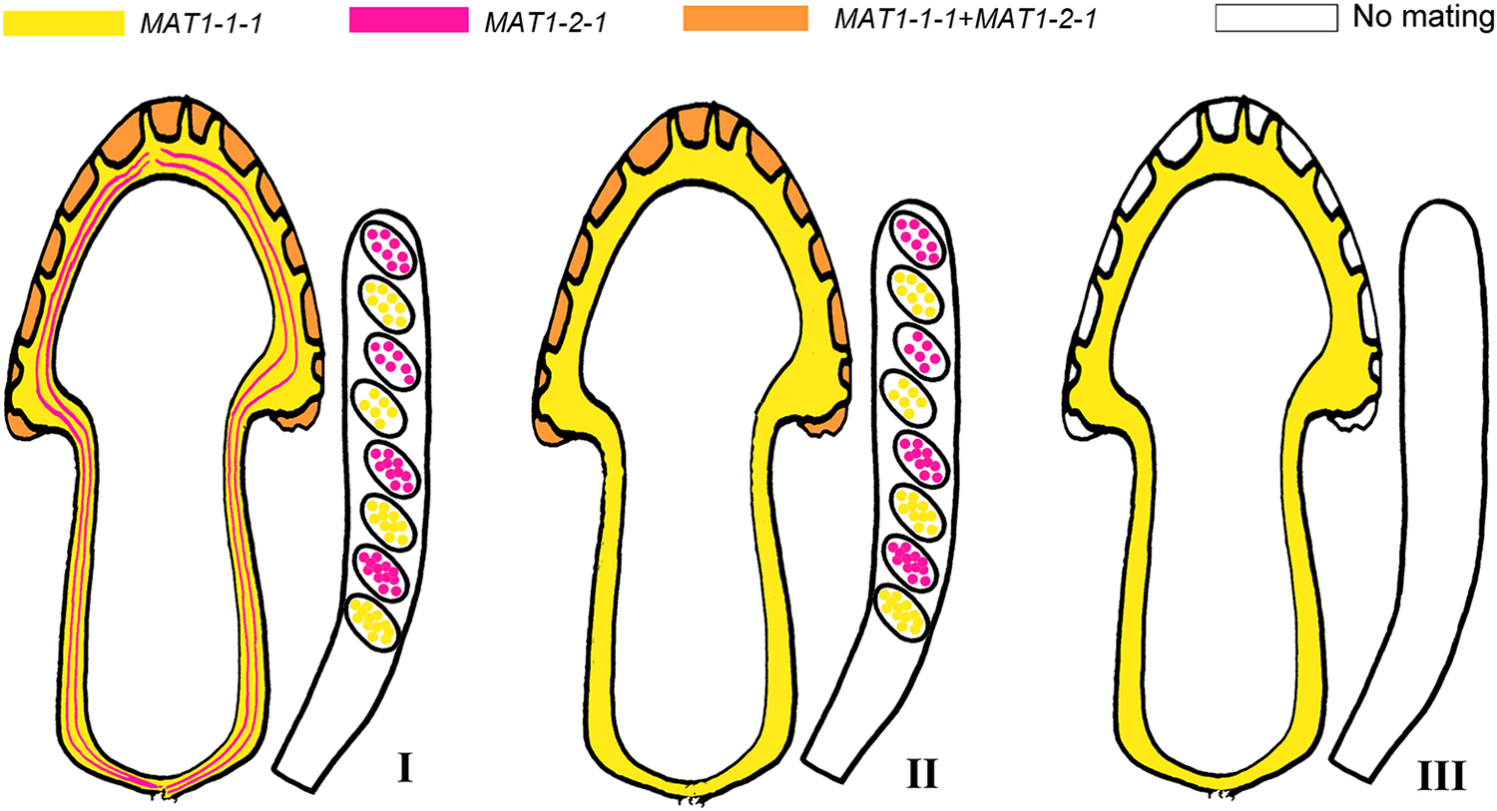
Schematic illustration of mating gene distribution in mature ascomata of morels. Although *MAT1-1-1* (depicted here in yellow) was detected much more dominant than *MAT1-2-1* (depicted in pink) in the sterile tissues (context of cap and stipe, and ridges on the cap), two mating types occurred equally in fertile tissues (hymenial layers, depicted in orange), and, thus, all 14 studied species must be heterothallic. The hyphae of the dominant *MAT1-1-1* served as ‘maternal’ tissue to support the whole ascomata, while *MAT1-2-1* served as ‘paternal’ partner to complete sexual reproduction. Three combinations were detected, both MAT loci are equally exhibited (I), *MAT1-1-1* acts as the dominant while *MAT1-2-1* plays a supporting role (II), and only a single MAT locus, exampled with *MAT1-1-1* here, is present and resulted in non-production of ascospores (no mating) (III). Occasionally, *MAT1-2-1* played ‘maternal’ while *MAT1-2-1* ‘paternal’, which is not illustrated here. Ascospores, when present, are mostly haploid homokaryotic multinuclear.

A total of 186 black morel ascocarps were harvested from twenty-five sites during the spring to autumn collection seasons of 2004–2016 (Table 1). To investigate the MAT distribution difference between fertile and sterile tissues, DNA from both the hymenia and stipes of each ascocarp was analysed, respectively representing fertile and sterile tissues, by PCR using primers specific for the *MAT* locus. Interestingly, the stipe usually harbors only a single mating type, either the *MAT1-1-1* or the *MAT1-2-1* gene, but hymenia always displayed both MAT-specific amplicons. Three spatial segregation types of mating types were identified, as shown in detail in Table 1 and Fig. 2. The first one was that both of mating types were found in hymenia and stipes (I); the second and also dominant one was that both mating types were detected in the hymenia, but only one mating type, either *MAT1-1-1* or *MAT1-2-1*, in stipes (II); the last one was that only one mating type was found in the ascocarps, which did not produce ascospores according to microscopic observation (III). By integrating the information from the analysis of ascospores, stipes and the hymenia, we interpreted the dominant component (sterile tissue, namely stipes etc.) of morel ascocarps was haploid, formed by one MAT mycelium, which was defined as ‘maternal’ (M) tissue to support the whole ascomata, and another putative genotype derived from the fertile tissue (hymenia) was defined as ‘paternal’ (P) partner to complete sexual reproduction and life cycle together with the maternal.

### Unequal MAT idiomorphs distribution in each species

In order to deeply investigate the presence and distribution of mating type genes of ‘the maternal tissue’ within and among the fourteen species, mating type genes detection of ‘the maternal tissue’ were conducted and assessed. Results indicated that, except *M. importuna* with the ratios of *MAT1-1-1*:*MAT1-2-1* around 1, all the other species were far more than 1, the highest one shown in *M. eximioides* even almost to 6. Though no rejection in these species, current results strongly indicated that *MAT1-1-1* was likely the dominant mating type of maternal tissue for morels ascocarps.

### Discrepant distribution of MAT idiomorphs between wild and cultivated populations of three species

The presence and spatiotemporal distribution of opposite mating types was evaluated in different tissues of ascocarps and in different species shown as above. Considering that several species among them have been successfully cultivated, as a control, cultivated ascocarps of *M. sextelata*, *M. eximia* and *M. importuna* were genotyped to investigate the opposite mating types existence and distribution and to be compared with their wild populations.

As expected, the single spores from cultivated ascocarps presented both mating types with approximately equal ratios, similar to the wild populations, indicating the frequencies of the two mating types occurred equally in single spores of wild and cultivated ascocarps in our study.

However, the distributions of MAT idiomorphs were shown to be discrepant between wild and cultivated ascocarps. Our results indicated that though those individuals belonged to the same species, rare type I (3.9%) was found in the wild populations of three species, whereas it was found to be dominant in the cultivated population. The stipe of grown morels always showed the presence of both MAT genes, in contrast, usually only one MAT detected in the stipes of wild ones. In conclusison, an inconsistent distribution of MAT exists between cultivated and wild morels of the same species.

## Discussion

Sequencing of the *M. eximia* genome paved the way and promoted the progress to characterize the MAT locus in some true morel species in this study. Further progress on understanding the life cycle and mating behavior of true morels is necessitated by the recent successful cultivation of several morel species. To date, their mating biology and conditions required for fruiting remains unknown, even for these successfully cultivated species. Thus, understanding the mating biology of them is critical both to conservation of natural populations as well as to development of artificial cultivation systems.

### Attainment of mating types and assessment of their genetic diversity and phylogeny

Notably, both mating type genes were first identified in fourteen black morel species after multiple attempts, including *M. tridentina*, *M. semilibera*, *M. sextelata, M. eximia*, *M. exuberans*, *M. importuna*, *Mel-*13, *Mel-*14*, M. eximioides*, *M. eohespera*, *M. purpurascens*, *Mel*-21, *M. dunalii* and *M. pulchella*, though they showed different length in both *MAT1-1-1* and *MAT1-2-1* nucleotide sequences. Analyses of MAT sequence showed high levels of nucleotide diversity (pi) in both *MAT1-1-1* (0.0488) and *MAT1-2-1* (0.0574) within these species, but low levels of nucleotide diversity (pi) within intraspecies as shown in Table 1. This indicated that MAT genes evolve rapidly among morel species but highly conserved within species. Strong purifying selection against deleterious mutations were suggested in MAT genes^11^, which probably resulted in low intraspecific polymorphism observed in MAT genes of morels. *MAT1-2-1* was found to be more variable than *MAT1-1-1* in the fourteen species. Differences in function, selective pressures, and expression levels of the two mating type genes may account for this difference^12–14^, and our analyses indicated different sexual competence between both mating types in morels, but functional and transcriptional assays for the *MAT1-1-1* and *MAT1-2-1* genes of morel species are needed in the future to assess the potential contribution of these factors.

Phylogenetic analysis of the *MAT1-1-1* and *MAT1-2-1* combined sequences matrix supported each of the fourteen species as a monophyletic group (Fig. 1) corresponding to the accepted species relationships within *Morchella* genus^15, 16^. Both MAT genes are recommended as good candidate markers for phylogenetic inferences in *Morchella* genus including those closely related species, as reported in other fungi^17–20^.

### Heterothallic reproductive modes of fourteen morel species

Fungal mating type systems play a pivotal role in controlling survival and sexuality. A better understanding of the reproductive modes that control fruiting in these morel species is therefore of both practical cultivation and economical relevance.

Here, we provide evidence to support the hypothesis that these fourteen species are heterothallic. Firstly, the findings that single spores from fourteen species harbor single opposite mating types with almost equal ratios strongly supports a heterothallic life style for each. Second, the presence of both mating type genes within the fertile tissue hymenia, paralleled with the dominant presence of only a single mating-type gene in the corresponding sterile tissue (type II), provides evidence of the heterothallic nature of these species and its prevalently haploid life cycle typical with other Ascomycota in which a maternal mycelium is fertilized by a paternal.

However, very unusually, haploid fruiting can occur without an opposite mating-type partner, given the existence of rare type III ascocarps (Table 1), where only one MAT gene was detected and no asci and ascospores in their mature hymenia were found after microscopic observation. It’s the first reported in *Morchella* genus to fruit without an opposite mating-type partner, which was also found in *Cordyceps militaris*, *Botrytis cinerea* and *Sordaria brevicollis*, where their haploid fruiting bodies were sterile and their progeny were usually undeveloped (no perithecia and ascospores)^14, 21–23^. They show a mixed mating system; i.e., individuals have the ability to outcross and haploid fruiting, though rare for the latter.

Our results supported that the fertile tissue (hymenia) of most morels carried both mating types, but, this contrasts with the expectations based on previous results where no heterozygosity was identified in many studies even when evaluated with codominant markers^2, 6, 7, 15, 16, 24^. Considering that these ascocarps were derived from outcrossing, as demonstrated by the presence of both mating types, till now, only difference at MAT locus was identified between their biparental genotypes. Usually, heterozygosity was expected in this case unless the fertilizing parent is the same as the maternal parent (homothallism or selfing) or the maternal and the fertilizing parents are closely related (inbred). Therefore, our current results suggest that these morels may have resulted from biparental inbreeding. Mating between closely-related parents has led to their progeny that may not display marker segregation except at the mating type locus^25, 26^. Our previous population genetic study is also consistent with this finding, which supported that inbreeding was prevalent in both *Mel*-13 and *M. eohespera* species^2^.

For the first time in *Morchella*, karyological staining in parallel with MAT screening among and within spores was conducted and have allow us to determine their haploid multinuclear homokaryotic level (Fig. 1). These nuclei in each spore are all copies of a single meiotic product and are not representatives of different meiotic products. But, it is still unknown what reasons lead to the production of aborted ascospores in each species or why fruiting bodies with no viable ascospores are produced.

Considering the haploid level occupying the predominant life cycle of morels, we suggested that heterokaryons are only transiently formed within a limited phase for the production of ascospores. The absence of heterokaryons during the dominant period of the morel life cycle implies that these species may contain a vegetative incompatibility system, as found in many other ascomycetes^27–29^. Genetic nonself recognition systems, probably work at the pre-fusion level, preventing the formation of anastomosis between strains to maintain the genetic integrity of each strain^29–31^ and protect resources within hyphae from exploitation by non-kin individuals during vegetative growth^27^. Moreover, the MAT locus was reported to be one of the loci governing vegetative incompatibility in *Neurospora crassa*, *Sordaria brevicollis*, and *Ascobolus stercorarius* ^32^, however, whether they play the same role and act on the vegetative incompatibility in *Morchella* spp. remains unknown. Also, the factors which mandate and underpin the transition from the vegetative to reproductive stages in morels still awaits to be uncovered. Additionally, an anamorphic phase of morels was recently described^33^ and also found in *M. sextelata*, *M. eximia*, *M. importuna* and several other cultivated species at their cultivation sites in China, however, what role asexual conidia played and whether act as fertilizing agents in morels are open questions.

### Orchestration of maternal and parental tissue with MAT segregation in morel ascocarps

In some ascomycetes, genetic material is usually typified with maternal and parental partitioning, and the dominant partner of the ascocarp is suggested to be the maternal one feeding the fruiting body^26, 34, 35^. Sexual identity, i.e., which mating type acts as male or female, is not defined. In order to determine whether both mating types play equivalent sexual roles in morel ascocarps, the stipes representing sterile part and hymenia representing fertile part were respectively detected for MAT diversity in this study.

Our results indicated that the hymenia of wild morels always displayed both MAT genes, whereas in the corresponding stipes a single mating type was always preferentially or exclusively amplified (Type II, Table 1). This provides definitive evidence for the following conclusions: 1) that most morels developed as a consequence of mating between strains of opposite sexual partners; 2) the stipes of morels, likely including other sterile tissue, resulted from homokaryotic/haploid mycelia and could be formed by mycelial strains harboring either *MAT1-1-1* or *MAT1-2-1*, behaving as ‘maternal’ partners in mating events; 3) both kinds of morel MAT mycelial strains are capable of producing the ‘maternal’-driven apothecial structures. We still did not find sexual structures (gametangia) in morels, but the current results indicated that a morel strain with either mating type might be hermaphroditic and have the capability of differentiating into ‘maternal’ and ‘paternal’ sexual structures.

However, for cultivated morels, based on the investigation in three cultivated species, not only their hymenia, but also their corresponding stipes always displayed both MAT genes (Type I, Table 1); this was greatly different from wild morels. Evidence of segregation of mating types among haploid single spores rejects the possibility of secondary homothallism and supports heterothallism. Given that our results above supported that competitions existed between both MAT mycelium in natural sites, we presumed the current special phenomenon might be related with adequate nutrition and optimal environmental conditions at cultivated sites, however the exact reasons remain unclear.

Additionally, we also found some ascocarps from natural sites, though this type was rare (Type III, Table 1), exhibited only one MAT gene and could not complete their life cycle, due to lacking of ascospores. This type, as we presumed above, was resulted from one unique mating behavior, sterile haploid fruiting.

### Spatial competition and unbalanced distribution of mating types in natural sites

Many studies carried out on the frequency distribution of mating types in natural field sites and revealed that, for some fungi, they are always reported to be patchy and unbalanced, such as *C. militaris* ^23^, *Cryptococcus neoformans* ^36^, *Mycosphaerella graminicola* ^13, 37^, *Phytophthora infestans* ^38, 39^, and *T. melanosporum* ^26, 29, 35^. In the current study, we monitored the distribution of morel strains with different mating types respectively from fourteen species in different morels grounds. Given that mother tissue discussed above usually were haploid and included one MAT, so we genotyped MAT of mother tissue (stipes) within different populations of different species to investigate the distribution and competition between both MAT mycelia.

The distribution analysis of strains with different mating types provides an intriguing scenario of morels. Firstly, based on the analysis of the total samples from each of the fourteen species, except *M. importuna* where *MAT1-1-1* was almost equal to *MAT1-2-1* which might be associated with many cultivated morels included, *MAT1-1-1* was detected significantly more commonly than *MAT1-2-1* in all the other species, especially in *M. eximioides* (almost 6:1), indicating a competitive advantage for *MAT1-1-1* strains, as raised in many studies^5, 26, 29, 37^. In some populations of these species, such as, Lvliang population in *Mel*-13, Changdu and Gannan populations in *M. eohespera*, unexpectedly extreme unbalance was observed where none of *MAT1-2-1* detected. Generally and apparently, MAT types frequency was skewed and unbalanced with dominantly more frequent *MAT1-1-1* than *MAT1-2-1*. Moreover, the diverse distribution types of mating types from wild and cultivated populations in this study indicated that a large-size population of strains is necessary.

Studies have shown, in some fungi, divergences in ecological, physiological, pathogenic or other functional importance are indicated between opposite mating types^5, 13, 40^, which likely affect the spatial distribution frequency of mating types in natural populations. Additionally, our results show clearly that MAT segregates equally in ascospores which means mating types should be in equal frequency in the population of ascopsores. However, *MAT1-1-1* is more common in the stipes than *MAT1-2-1*, indicating that *MAT1-1-1* is more competent to play a female sexual role. This might suggest mating types in *Morchella* genus have become more like “sexes” than the typical fungal mating types.

In China, morels are successfully cultivated in many sites, but the biggest problem growers face is the unstable production. No prior detection and screening of mating types during the strains breeding processs might be one of the reasons. We speculated the predominance of one MAT may be important as a reason for degeneration of strains due to strains with a particular mating type competing to survive. The spatial segregation of mating type could decrease the probability of sexually compatible partners meeting, lower the odds of mating and eventually lead to declining production.

**Figure 3:**
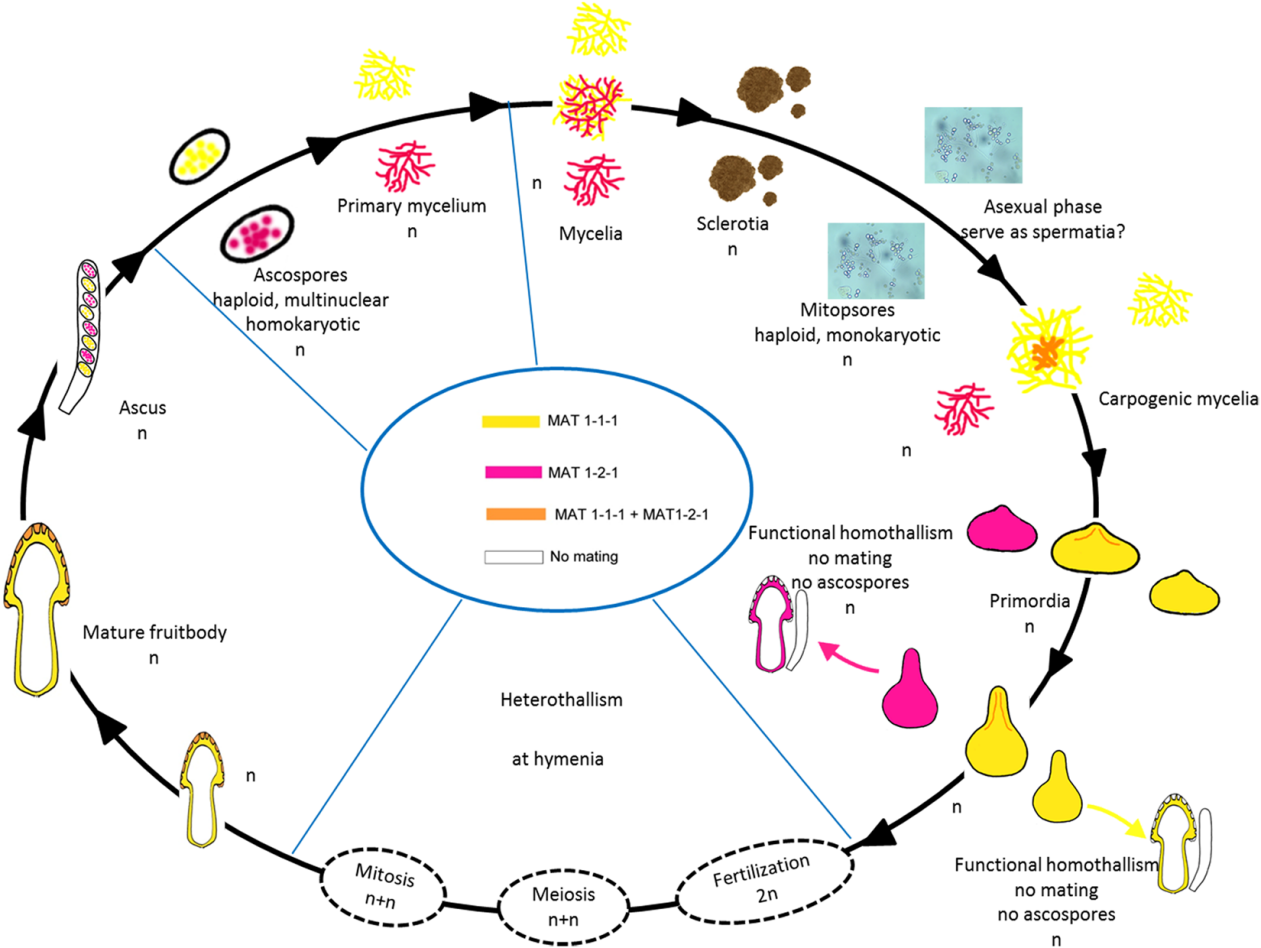
Proposed life cycle of the genus *Morchella*. Here, we illustrated two kinds of life cycles identified in morels, viz. haploid fruiting and heterothallism. Considering two types (I and II, shown in Fig. 2) were found in their heterthallism life cycle, we took type II with MAT1-1-1 serving as maternal tissue for example in this figure.

Finally, here, we use molecular markers and current results to reconsider the potential morels life cycle (Fig. 3). Heterothallism is shown here to be the dominant reproductive mode in all of these fourteen species, with low proportional sterile haploid fruiting. However, the morphology of the fertilization process in the morel life cycle still remains elusive.

### Conclusions and perspectives

To the best of our knowledge, this is the first study to determine mating types of morels and to assess their distribution in nature and in cultivation. The selection of morel strains, without clearly characterized genetic background, are prone to producing fruiting bodies susceptible to degeneration, ultimately resulting in wasted human resources and time. To avoid such problems in production efforts, the prior screening and determination of mating types with the aid of molecular markers is the important part of morel cultivation. The methods and results presented in this study could effectively facilitate selection, domestication, and management of strains to decrease the possibility of degeneration and also provide a theoretical reference for the artificial cultivation of morels and even the industrial-scale production in the future.

## Material and Methods

### Obtainment of mating type genes

Based on the genome sequence of *Morchella eximia*, *MAT1-1-1* and *MAT1-2-1* were identified using BLASTn and BLASTx against the NCBI nucleotide and protein database by sequence similarity searches (http://www.ncbi.nlm.nih.gov/BLAST/).

### Sampling

168 wild samples collected in 25 populations of 10 provinces in China from 2004 to 2016; 10 wild samples from France; 5 wild samples from Cyprus; and an additional from Pakistan were used here to represent as wide geographical differences as possible. Based on the multi-gene phylogenetic species recognition results^2, 16^, the above 186 specimens belong to 14 black morel species, namely *M. tridentina*, *M. semilibera*, *M. sextelata, M. eximia*, *M. exuberans*, *M. importuna*, *Mel-*13, *Mel-*14*, M. eximioides*, *M. eohespera*, *M. purpurascens*, *Mel*-21, *M. dunalii* and *M. pulchella*. Additionally, 39 cultivated samples from five growth sites of *M. sextelata*, *M. eximia* and *M. importuna* were included here in order to detect if the ratio of mating types is consistent with a theoretical ratio of 1:1 (*MAT1-1-1*:*MAT1-2-1*) between wild and cultivated populations. Detailed species and geographical information of these 223 samples are presented in Table 1.

### Single spore isolates and cultural conditions

Fifty single ascospores were randomly isolated from each species. Ascospores were washed, suspended in sterilized water, and 200 μL of a solution adjusted to a concentration of 200–300 ascospores mL^−1^ was spread on potato dextrose agar (PDA) and incubated at 23–25 °C for 1–2 d. Single germinated ascospores were picked using a dissecting needle under a dissecting microscope (Zeiss 455094) and transferred to a new PDA Petri dish, which was incubated at 23–25 °C for 1 week.

### DNA extraction

#### DNA extraction from single spore cultures

A culture plug bearing actively growing mycelia was transferred onto freshly-prepared PDA. When the growth of the fungus reached the edge of the plate, mycelia were scraped from the surface with a sterilized inoculating shovel and transferred into a 1.5 ml microcentrifuge tube. Samples were immediately frozen in liquid nitrogen and stored at −80 °C until used.

#### DNA extraction of fruiting bodies

To detect whether there are differences in mating type genes between fertile tissue and sterile tissue, the hymenia and stipe of each ascomata were separated for DNA extraction to respectively represent fertile and sterile tissue and also used for comparison of different individuals within and among species. But, for *M. dunalii*, we just got some pieces of tissue from the hymenia of samples, so, no stipes DNA could be extracted and no analysis on MAT distribution of stipes in this species could be conducted.

Samples (mycelia/hymenia/stipe) were ground to a fine powder in a 1.5 ml microcentrifuge tube using a Kontes pellet pestle (Kaimu, China). Once pulverized, the samples were suspended in 700 μl of CTAB extraction buffer (100 mM Tris–Cl pH 8.4, 1.4 M NaCl, 25 mM EDTA, 2% CTAB), and incubated for 1.5–2.0 h at 65 °C, during which time they were gently inverted 3–5 times. After the samples were cooled to room temperature, 700 μl of chloroform-isoamyl alcohol (24:1) was added to each tube. The mixture was vortexed briefly, centrifuged at 12,000 g for 10 min, and then 500 μl of the upper phase was carefully transferred to a new 1.5 ml microcentrifuge tube. After a second chloroform-isoamyl alcohol (24:1) extraction was performed, the supernatant was transferred to a new 1.5 ml microcentrifuge tube and an equal volume of 100% isopropanol at 20 °C was added to each tube. The tube contents were mixed briefly by inversion to obtain a homogeneous solution and then they were stored overnight at 20 °C to precipitate total genomic DNA. After the tubes were warmed to room temperature they were centrifuged at 12,000 g for 10 min and the supernatant was discarded. The DNA pellet was washed consecutively with 70% and 100% ethanol, air-dried and then resuspended in 100 μl of sterile double distilled H2O. All genomic DNA samples were stored frozen at 20 °C until ready for use.

### MAT PCR amplification

The mating type-specific primers for *MAT1-1-1* and *MAT1-2-1* genes were designed according to morel genome sequencing. The primers’ sequences are reported in Table S1 and used for the following PCR and DNA sequencing. Each PCR reaction contained 1 μl of 20 ng/μl genomic DNA, 2.5 μl of 10 × PCR reaction buffer, 0.5 μl dNTP mix (10 mmol), 2 μl each of primers (5 μmol), 1.5 μl bovine serum albumin (20 mg/ml) and 1.5 U of Taq DNA polymerase (Biomed, China). The final volume was adjusted to 25 μl with sterile distilled H_2_O. PCRs were conducted in an Applied Biosystems 2720 thermocycler (ABI, Foster City, CA), using the following cycling parameters: 94 °C for 3 min, 35 cycles of 94 °C for 1 min, 50 °C for 30 s, 72 °C for 1 min, followed by a final extension of 10 min at 72 °C. Amplicons were electrophoresed in 1.2% agarose in 1 × TAE, stained with GoldView™ (Guangzhou Geneshun Biotech Ltd., Guangdong, China), and then photographed over a ultraviolet transilluminator. PCR products were purified using a Bioteke DNA Purification Kit (Bioteke Corporation, Beijing, China), sequenced with ABI BigDye ver 3.1 (Sangon Co., Ltd., Shanghai, China), and then run on an ABI 3730 DNA Analyzer. The raw DNA sequences of *MAT1-1-1* and *MAT1-2-1* genes amplified were aligned with SeqMan (DNAStar Package, Madison, WI). Sequences generated in the present study have been deposited in GenBank under accession numbers KY508074-KY508239.

### Mating type detection and screening for single ascospores, hymenia and stipes

According to the current results, the sequence length of *MAT1-1-1* and *MAT1-2-1* are respectively 729–736 bp and 398–408 bp, so mating type gene detection of the 700 single ascospores, 223 hymenia and 218 stipe cultures could be performed by observing amplicon length over a ultraviolet transilluminator after electrophoresis for first screening. This method greatly reduced the sequencing cost and accelerated mating type detection. Then, some samples in each species (totaled eighty-three samples) were chosen for sequence analysis of their *MAT1-1-1* and *MAT1-2-1* genes for genetic diversity and phylogenetic analysis.

### Sequence diversity of *MAT1-1-1* and *MAT1-2-1* and phylogenetic analysis

*MAT1-1-1* and *MAT1-2-1* datasets were respectively aligned automatically with MAFFT^41^, manually adjusted using BioEdit ver. 7.0.9 ^42^ (http://www.mbio.ncsu.edu/bioedit/bioedit.html) and then manually trimmed to eliminate uneven ends. Nucleotide diversity and variable sites were estimated for each gene using DnaSP^43^. Maximum parsimony (MP) bootstrap analyses were conducted for the two-locus, *MAT1-1-1* and *MAT1-2-1* matrices.

MP analyses were performed in PAUP* V 4.0b10 ^44^ based on a heuristic search of 1,000 replicates with random stepwise addition using tree-bisection-reconnection branch swapping and starting with trees obtained by the stepwise addition of sequences. All of the characteristics were equally weighted. Parsimony bootstrap analyses with 1,000 replicates were used to assess clade support.

### Ratio assessment of mating types and Statistical analysis

The presence ratios of both mating types were assessed respectively for intra and inter-species single spore isolates, and among hymenia and stipes of intra and inter-species. The mating type ratios within and among species, and between wild populations and cultivated populations were compared. The Fisher’s exact test using SPSS software version 15.0 (SPSS, Inc., Chicago, IL, USA) was conducted to determine whether the ratio of mating types (*MAT1-1-1*:*MAT1-2-1*) did not deviate from a theoretical ratio of 1:1.

### Karyological analysis

Ascospores were stained with DAPI (4′-6-diamidino-2-phenilindole) to visualize nuclei. Pools of purified ascospores were incubated for 15 min in the staining reagent (4 mM DAPI, 100 mM Tris-HCl pH 7.5, and 20% glycerol) directly on the microscope slide. Nuclei were observed under fluorescent light using an Olympus Fluoview FV1000 laser-scanning microscope.

## Electronic supplementary material

Supplementary Info 1
