# Supplementary material for: Mixed-reproductive strategies, competitive mating-type distribution and life cycle of fourteen black morel species
Source: Sci Rep. 2017 May 4;7:1493. doi: 10.1038/s41598-017-01682-8 (PMC5431422; doi:10.1038/s41598-017-01682-8)
Supplement: Supplementary file 1 — Supplementary Info 1 [file 41598_2017_1682_MOESM1_ESM.doc]

**Mixed-reproductive strategies, competitive mating-type distribution and life cycle of fourteen black morel species**

Xi-Hui Du^1^ Qi Zhao^1^ En-Hua Xia^2^ Li-Zhi Gao^2^ Franck Richard^3^ Zhu L. Yang^1^*

^1^Key Laboratory for Plant Diversity and Biogeography of East Asia, Kunming Institute of Botany, Chinese Academy of Sciences, Kunming, China, ^2^Plant Germplasm and Genomics Center, Germplasm Bank of Wild Species, Kunming Institute of Botany, Chinese Academy of Sciences, Kunming, Yunnan 650201, China, ^3^UMR 5175 CEFE, INSERM, Campus CNRS, F-34293 Montpellier, France

***Correspondence:**

Prof. Dr. Zhu L. Yang

Address: 132#, Lanhei Road, Panlong District, Kunming, China

E-mail: [fungi@mail.kib.ac.cn](mailto:fungi@mail.kib.ac.cn)

**Table S1.** PCR and sequencing primers.

| **Locus** | **Primer** | **Reference** | **Sequence (5’-3’)^b^** |
| --- | --- | --- | --- |
| MAT1-1 |  |  |  |
|  | MAT11L | This study | CCACCTTCTGAGTCCATTAT |
|  | MAT11R | This study | GTTATTCTCGACAAGGTGTG |
| MAT1-2 |  |  |  |
|  | MAT22L | This study | TTATTAGACCATGTTCCTCG |
|  | MAT22R | This study | CAGTATTATCACCAACCGTA |
